# Identification and growth-promoting effect of *Paecilomyces lilacinus* a biocontrol fungi for walnut rot disease

**DOI:** 10.1371/journal.pone.0314160

**Published:** 2024-12-06

**Authors:** Zhi-Jin Liu, Lu-Lu Shan, Xiao-Fei Chen

**Affiliations:** Tarim University/Key Laboratory of Integrated Pest Management (IPM) of Xinjiang Production and Construction Corps in Southern Xinjiang, College of Agriculture, Alar, China; ICAR - Indian Agricultural Research Institute, INDIA

## Abstract

*Cytospora chrysosperma* is the primary pathogen responsible for walnut rot disease, affecting a wide variety of hosts. Currently, chemical agents, particularly agricultural Fungicides, are commonly utilized for the prevention and management of walnut rot. However, this practice has led to the development of drug-resistant pathogens, complicating disease control efforts. This study aimed to propose a safer and more efficient biocontrol strategy for combating walnut rot disease. The research focused on targeting *C*. *chrysosperma*, and through cross-confrontation methods, an antagonistic strain named *Paecilomyces lilacinus* 5–38 was isolated from walnut rhizosphere soil, exhibiting a significant inhibitory effect on *C*. *chrysosperma* with an inhibition rate of 78.71%. Results indicated that strain 5–38 not only demonstrated effective control but also displayed a broad-spectrum antifungal activity. The study also investigated the impact of different concentrations of antagonistic fermentation filtrate on *C*. *chrysosperma* mycelia, revealing a stronger inhibitory effect with increasing concentrations and a degree of thermal stability. Pot experiments demonstrated that a concentration of 150 mg/ml of antagonistic fermentation filtrate enhanced seed germination rates and various growth parameters of walnut seedlings, including seedling height, root length, root number, leaf area, and dry weight. Specifically, seedlings treated with *P*. *lilacinus* 5–38 showed significant increases of 30.12%, 33.89%, 81.89%, 6.83%, and 98.26% in these respective parameters. This study represents the first documentation of *P*. *lilacinus* as a promising biological agent for walnut rot control.

## Introduction

Walnut (*Juglans regia* L.) is a significant economic tree species within the walnut genus Pidaceae and ranks as one of the world’s four major dried fruits, boasting rich nutritional and medicinal value [1]. Xinjiang stands as the second-largest province for walnut cultivation in China, with a planting area that reached 4.14x10^5^hm^2^ and an annual output of 1.15x10^6^t in 2020 [2]. However, in recent years, the extensive walnut planting in Xinjiang has been plagued by walnut rot disease, significantly impeding the healthy growth of the walnut industry in the region [3–5]. Presently, disease management relies heavily on chemical control methods, but these chemical fungicides pose various issues, including environmental pollution, risks to human and livestock safety, and pesticide residues [6,7]. Therefore, the development of efficient biological and fungal strains to replace chemical agents in controlling walnut rot disease can not only effectively manage disease occurrence and progression but also mitigate the problems associated with chemical control.

*Paecilomyces lilacinus* widely distributed throughout the world, it has the advantages of high efficacy, wide host, easy to cultivate and so on, especially in the control of plant pathogen nematodes [8]. Related studies have confirmed that *P*. *lilacinum* has antagonistic activity against a variety of plant pathogenic fungi, bacteria and even viruses [9–12]. In the study of the control mechanism of plant fungal diseases, chitinase, β-1,3 glucanase and other related cell wall degrading enzymes have been proved as defense enzymes with disease resistance. The mechanism of action is to degrade the pathogenic fungal cell wall, thus killing the pathogen [13,14]. Currently, there is a lack of research on the antagonistic and growth-promoting impacts of *P*. *lilacinus* on walnut rot disease. This study aims to investigate the inhibitory properties of secondary metabolites produced by *P*. *lilacinus* against *C*. *chrysosperma*, focusing on disease prevention and growth enhancement. Additionally, the research will explore the stability of indoor antagonistic fermentation filtrate and the potential of using *P*. *lilacinus* for controlling various fungal diseases in crops.

## Materials and methods

### Materials used in the experiment

In May 2023, the test soil was collected the walnut orchard in Wensu County, Aksu Region. The walnut variety in the orchard was "Wen 185", the age of the trees was 30 years, and the spacing between the rows was 5 m×7 m. The walnut trees with uniform growth were selected for sampling, and 0~5 cm of topsoil was removed from the main trunks of the walnut trees at a distance of 1 m. The soil samples were taken from the soil auger at a distance of 5–20 cm, and then the soil samples were put into a sterile bag, and stored in a refrigerator at 4°C for use.

The following pathogenic fungi were provided by the Key Laboratory of Integrated Pest Management in Southern Xinjiang Corps:*Cytospora chrysosperma 、 Cytospora nivea* 、*Valsa mali*、*Valsa ambiens*、*Cytospora chrysosperma*、*Cytospora leucostoma*、*Alternaria alternata*、*Verticillium dahliae*、*Fusarium oxysporum*.

The test walnut seed variety is "Wen 185", the weight of a single fruit is not less than 12g, and the kernel is full, free of mildew and insect pests.

### Isolation of soil microorganisms

A soil sample weighing 10.0 g was placed into a conical flask along with 90 mL of sterile water and glass beads. The mixture was shaken on a shaker for 30 minutes to create a suspension of the soil sample. This suspension 10ml suspension was taken and serially diluted to 10^−2^, 10^−3^ and 10^-4^.Subsequently, 100 μL of each diluted solution was absorbed onto PDA medium for strain isolation. The plate was incubated at 26°C for 3–7 days. The strain was then subcultured to purify it, labeled and stored at 4c°Cfor later use.

### Screening of the antagonistic fungi

Using the plate confrontation culture method,a cross was made at an equal distance (approximately 2.5cm) from the pathogen on the PDA plate inoculated with *C*. *chrysosperma*. The control consisted only of the pathogen, with each fungus treated in three replicates. The cultures were maintained at a constant temperature of 26°C for 3days. Once the control mycelium was close to filling the plate, colony morphology was observed, and colony diameter was measured to calculate the inhibition rate of colony growth. The inhibition rate was calculated using the formula:

Inhibition rate (%) = [(colony diameter ofthe control group—colony diameter ofthe test group)/ colony diameter ofthe control group]×100%.

### Identification of the antagonistic fungal

#### Morphological characteristics

The preserved strains were incubated for 3 days on PDA medium at 28°C. During this period, colony characteristics such as growth rate, mycelium texture, separation, thin density, presence of exudate, and absence of pigment were observed and recorded.

Morphological observation of mycelium and conidia was conducted by inserting a sterilized cover slide obliquely into the PDA medium plate, mycelium morphology under a microscope. Visual fields with typical features were photographed and recorded for identification of the antagonistic genus following the fungi Identification Manual [15].

#### Molecular biology identification

A small number of hyphae was carefully selected from the activated plate using a sterilized inoculation needle and transferred into 100 ml of liquid PDA medium. The sample was then placed in a 28°C, 180 r/min shaker for 3–5 days (Speed-regulating mini centrifuge SMD BioEngineering (Shanghai) Co., Ltd). Subsequently, it was filtered on a superclean bench, rinsed three times with sterile water, drained using sterile filter paper, and finally placed in a 45°C-drying box for 4 hours(Electric heating constant temperature blast drying box DHG-9240AS Ningbo Jiangnan Instrument Factory). The dried hyphae were then transferred into a sterilized mortar and pestle and ground with liquid nitrogen added in small increments. Genomic DNA extraction of the test strain was performed using the Ezup column fungal genomic DNA extraction kit (Shanghai Biological Engineering Co., Ltd.), followed by storage in a 4°C refrigerator after electrophoresis in 1% agarose gel.

The extracted strain DNA was used as a template with the fungal universal primers ITS 1 (5’-TCCGTAGGTGAACCTGCG-3’) / ITS 4 (5’-TCCTCCGCTTATTGATATGC-3’) [16]. The amplification reaction was carried out in a 50 μl system consisting of 2 μl DNA template, 2 μl each of upper and lower primers, 25 μl Taq PCR Master Mix, and 19 μl ddH2O. PCR amplification conditions included predenaturation at 95°C for 5 min, denaturation at 95°C for 35s, annealing at 55°C for 35s, extension at 72°C for 2 min by 35 cycles, and extension at 72°C for 10 min. The amplified products were analyzed by 1% agarose gel electrophoresis and viewed using an integrated gel imager. The PCR amplification productes were sent to Shanghai Biological Engineering Co., LTD. A phylogenetic tree based on ITS genes was constructed using MEGA 11 software to determine the taxonomic status of the antagonistic strains.

#### Inhibition of the antagonistic strains against eight pathogenic fungi

In this study, the walnut tree rot pathogen *C*. *nivea*, the apple tree rot pathogen *V*. *mal*i, the fragrant pear tree rot pathogen *V*. *ambiens*, the elegans tree rot pathogen *C*. *chrysosperma*, the almond tree rot pathogen *C*. *leucostoma*, and the walnut tree brown spot pathogen *A*. *alternata*, *V*. *dahliae*, and *F*. *oxysporum* were selected as target fungus. Antagonistic strains were positioned 2.5 cm away from the pathogenic fungus in a symmetrical manner, with PDA culture medium inoculated with pathogen cake alone serving as the control. Each treatment was replicated three times. The plates were then incubated at a constant temperature of 26°C. Following growth of colonies in the control group, The inhibition rate was calculated using the formula:

Inhibition rate (%) = [(colony diameter ofthe control group—colony diameter ofthe test group)/ colony diameter of the control group]×100%.

#### Effects of different concentrations of antimicrobial fermentation filtrate on *C*. *chrysosperma* hyphae

The fermentation filtrate of 3%, 6%, 9%, 12%, 15% concentration is mixed with the ratio of PDA (1:1).The mixture was then poured into a petri dish, with the walnut rot pathogen inoculated in the center of the plate. A PDA plate without fermentation filtrate was used as the blank control. The cultures were maintained at a constant temperature of 26°C, with each treatment being replicated three times. Once the control fungus had filled the Petri dish, the colony diameter was measured using the cross method to calculate the inhibition rate of the fermentation filtrate on mycelium. Following growth of colonies in the control group, The inhibition rate was calculated using the formula:

Inhibition rate (%) = [(colony diameter ofthe control group—colony diameter ofthe test group)/ colony diameter of the control group]×100%.

#### Determination of the thermal stability of filtrate

Five centrifuge tubes containing 10 ml of fermentation filtrate were placed in water baths at temperatures of 55°C, 65°C, 75°C, 85°C, and 95°C for 30 minutes. A positive control was prepared by mixing fermentation filtrate without temperature treatment with PDA culture medium in a 1:3 ratio. Another control was set up using only PDA culture medium without the addition of fermentation filtrate. The mixture was poured onto plates and allowed to solidify. Pathogenic fungi were placed in the center of the plates. Each treatment was replicated three times and incubated in the dark at 26°C until the pathogenic fungus hyphae in the control group had grown over the plate. Subsequently, the diameter of the pathogenic fungus colonies was measured, following growth of colonies in the control group, The inhibition rate was calculated using the formula:

Inhibition rate (%) = [(colony diameter ofthe control group—colony diameter ofthe test group)/ colony diameter ofthe control group]×100%.

#### The effect of biocontrol fermentation liquid on walnut rot spot

Branches were collected from healthy and uniform 1–2 year old walnut trees, cut into 10–15 cm segments, washed with tap water, disinfected with a 0.6% sodium hypochlorite solution for 15–20 minutes, rinsed with sterile water 3–4 times until no odor of sodium hypochlorite was detected, and left to naturally dry at room temperature. The branches were then sealed on both ends with melted paraffin using an alcohol lamp to retain moisture, and left to dry.

#### Independent branch spot control test

Punch holes in the branches with a sterilized 5 mm diameter hole punch, then inoculate each hole with a 5 mm diameter *C*. *chrysosperma* fungus cake (mycelial side facing down). Apply one inoculation point per branch. After cultivate at a constant temperature of 26°C for two days, remove the pathogenic fungi using an inoculation needle. Dip a brush into 3% amount of antagonistic fungi fermentation liquid and apply it to the branches three times (allowing to air dry between each application). After moisturizing and cultivating at 26°C for 15 days, observe and measure the lesion areas. Use *C*. *chrysosperma* as a control, repeat the experiment three times, and calculate the control effect.

Disease spot area (cm^2^) = 1 / 4 π long diameter and short diameter.

Prevention effect (%) = (control spot area-treated spot area) / control spot area ×100.

### Effect of strain fermentation filtrate on the growth of walnut seeds

#### Pretreatment of shelled walnut seeds

The shelled walnut seeds were gently split with a walnut clip, and the walnut kernel contacted the antagonistic fungi fermentation filtrate. At five concentrations of 50 mg/ml, 150 mg/ml, 250 mg/ml, 350 mg/ml and 450 mg/ml, the culture medium without the fermentation filtrate served as the control (CK), with each treatment consisting of 25 walnut seeds repeated 5 times.

#### Seed soaking and sprouting treatment

The prepared walnut seeds were surface disinfected with 75% ethanol for 30s, rinsed in sterile water for three times, and dried naturally.

Seed treatment of the test group the shelled walnut was gently split using a walnut clip to avoid the walnut kernel from unable to touch the antagonistic fungi fermentation filtrate. Walnut seeds were individually soaked in five concentrations of fermentation filtrates for 24 h. After soaking, the seeds were rinsed with sterile water and placed in a 28°C incubator to stimulate germination. The germination rate was counted before sowing on the hole plate.After germination, seeds were sown in a hole plate (32 holes, 6 cm 4.5 cm), 2–3 seeds per hole, covered with l cm thick matrix (vermiculite: perlite: peat: soil volume ratio was 1:1:1:1), germination rate and rotten seed rate were counted after germination and true leaves.Control seed treatment liquid medium without antagonistic fungi was used as control for 24 h.

Germinatio rate /% = (number of white seeds after germination / number of tested seeds)×100; Germination rate /% = (total number of germinated grains / numbers of tested seeds) ×100;

Rotten seed rate /% = [(number of pests + decay number) / number of tested seeds] ×100.

#### Effect of strain fermentation filtrate on the growth of walnut seedlings

A potted experiment on growth promotion of walnut seedlings was conducted at the Key Laboratory of Southern Xinjiang Integrated Pest Management Corps of Tarim University. The experimental materials consisted of dried fruits with full kernels, no mold or insects, and a single fruit weight of 12 g or more, which were selected and naturally dried. The control group received sterile water treatment, with each treatment being repeated 5 times, using 25 seeds per treatment and 5 seeds per replication. Prior to soaking, the walnut shells were opened to allow contact between the fermentation filtrate and the walnut kernels. After soaking the seeds for 1 day, they were washed with clean water, placed in a moist germination box, and subjected to germination treatment in a 30°C constant-temperature incubator. Seed germination was monitored daily, with any rotten or moldy seeds promptly removed. Once the seeds germinated, they were planted in flowerpots (20 cm × 20 cm), with 1 seed per hole, and covered with a 1 cm thick substrate (vermiculite: perlite: peat: soil volume ratio of 1:1:1:1) in three replicate groups.

Indoor potted seedlings treatment involved sowing walnut seeds treated with germination on flowerpots, followed by watering with antagonistic fungi fermentation filtrate (test group received 150 mg/ml fermentation filtrate, while control group received sterile water) at 50 ml per point every 5 days. After 20 days of seed growth, the indoor potted seedlings were transplanted into outdoor field pots.

In July 2023, potted walnut seedlings will be transplanted into a 40 cm diameter pot at Tarim University’s water-saving irrigation field. The seedlings will be watered three times with 500 ml of fermentation filtrate every 10 days. After 90 days of growth post-transplanting, in September 2023, five seedlings will be randomly selected for measuring dry weight, height, root length, root number, and other biological indicators. It is important to ensure the integrity of the plants when pulling out the walnut seedlings and to wash the roots slowly with sterile water to maintain their integrity.

#### Data analysis

Multiple comparisons of test data were implemented by Duncan’s new multiple range testusing sPss version 16.0 software.

## Results

### Isolation and screening of biocontrol strains

**Fig 1:**
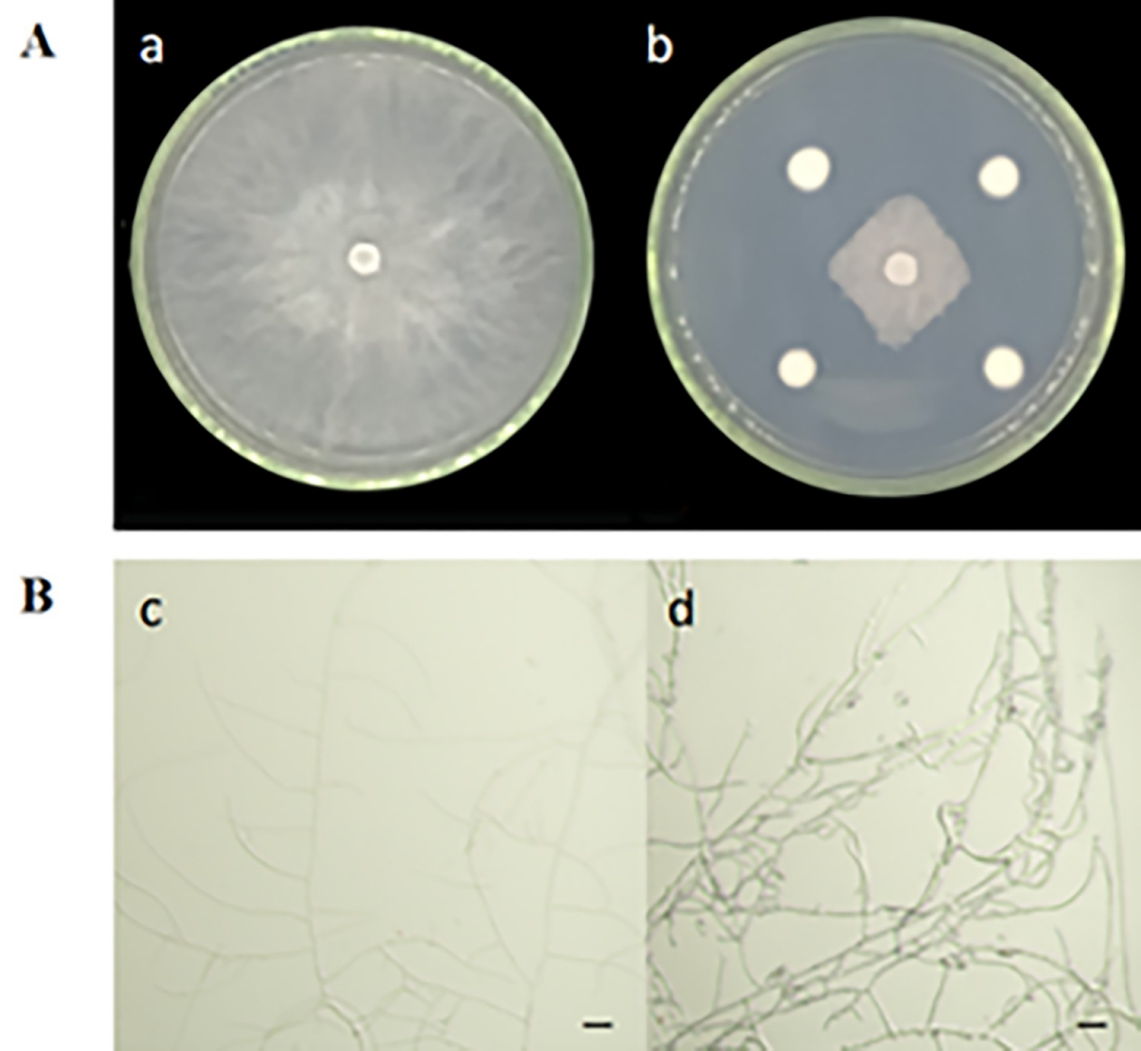
The antibacterial efficacy of the antagonistic strains against *C*.*chrysosperma*. A: Initial screening results of antagonistic strains; B: Antimicrobial effect of antagonistic strains on *C*.*chrysosperma* a: Screening control; b: Antimicrobial effect of strains 5–38 against *C*.*chrysosperma*; c: Pathogen hyphal control; d: *C*. *chrysosperma* hyphal morphology after strain 5–38 confrontation; scale = 20 μm.

A total of 293 fungi were isolated using the dilution coating plate method, with 34 strains exhibiting antagonistic effects on *C*.*chrysosperma*. Among these, 7 strains demonstrated an antimicrobial rate exceeding 70% (Table 1). The strains with over 70% inhibition were subjected to further screening (Table 2). Among them, strain 5–38 displayed the most potent inhibitory effect on *C*.*chrysosperma* (Fig 1 Ab), achieving an inhibition rate of 78.71%. Observation of the hyphae inhibition revealed localized expansion and rupture of the pathogenic fungi by strain 5–38, along with a deepening of mycelium color and inhibited hyphal growth (Fig 1Bd). Consequently, strain 5–38 was chosen for subsequent studies in this research.

**Table 1:** Preliminary screening results of antagonistic fungi.

Strains	Colony diameter (cm)	Inhibition rate (%)	Strains	Colony diameter (cm)	Inhibition rate (%)
A-01	2.95±0.13	63.13±1.65	5–35	2.42±0.13	69.75±1.72
A-02	3.63±0.13	54.63±1.66	5–38	1.78±0.03	77.66±0.40
3T-01	2.57±0.08	67.92±1.06	20–48	1.99±0.06	75.13±0.80
3T-03	3.00±0.12	62.47±1.61	20–57	2.11±0.15	72.74±2.58
3T-05	3.88±0.07	51.50±0.95	20–59	3.15±0.05	60.62±0.63
3T-09	2.66±0.08	66.70±1.01	20–61	3.35±0.17	58.13±2.18
3T-34	5.10±0.13	36.25±1.70	9T-13	3.52±0.13	56.00±1.71
3T-37	2.53±0.15	68.32±1.83	AKS-05	2.41±0.13	69.90±1.65
3T-38	4.20±0.19	47.50±2.42	AKS-07	2.46±0.19	69.28±2.38
3T-45	2.54±0.13	68.24±1.70	AKS-11	2.63±0.11	67.08±1.38
3T-50	3.07±0.07	61.67±0.95	WS-03	3.04±0.22	61.96±2.76
3T-51	2.38±0.20	70.29±2.50	WS-04	2.57±0.10	67.91±1.37
3T-52	2.24±0.12	71.96±1.58	WS-10	2.24±0.23	71.95±2.84
3T-59	4.50±0.16	43.75±2.03	WS-15	4.83±0.17	39.63±2.06
3T-64	2.43±0.18	69.67±2.50	WS-27	2.42±0.08	69.70±1.04
5–05	2.98±0.14	62.70±1.75	WS-34	2.13±0.13	73.41±1.60
5–11	4.30±0.15	46.25±1.56	WS-38	3.54±0.17	55.75±2.13

Note: The data listed in the table are average ± standard deviation.

**Table 2:** Rescreening results of antagonistic strains.

Strains	Inhibition rate (%)	Strains	Inhibition rate (%)
3T-51	69.50±0.82bc	20–57	70.87±2.49bc
3T-52	70.50±1.88bc	WS-10	69.37±0.62bc
5–38	78.71±0.93a	WS-34	71.79±1.42bc
20–48	72.54±3.35b		

Note: The data listed in the table are mean ± standard deviation, and different lowercase letters represent whether the difference is significant in the <0.05.

### Identification of fungus

#### Morphological identification

**Fig 2:**
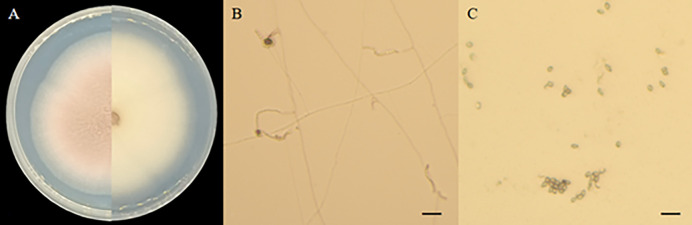
Morphological characteristics of strain 5–38. A: Colony front and back; B: Mycelial and conidium; C: Conidiospore; Scale = 20μm.

On PDA medium, strains 5–38 initially displayed white colony color, with mycelium spread evenly in a radial direction, round colony, loose cotton texture and wheel pattern. These strains exhibited slow growth at a rate of 0.36cm/day. As strain 5–38 produced spores, the front of the colony transitioned from white to pink. The colony darkened with an increase in spores, while the back remained light yellow (Fig 2A). The conidia were nearly round and transparent, with thick conidial stems in a bottle shape, rounded ends, or short branches. The conidia formed monospore chains, while the mycelium appeared transparent and slender (Fig 2B and 2C). Based on morphological characteristics and the Manual of Fungi Identification, it was initially classified as *Paecilomyces*.

#### Molecular biological identification

**Fig 3:**
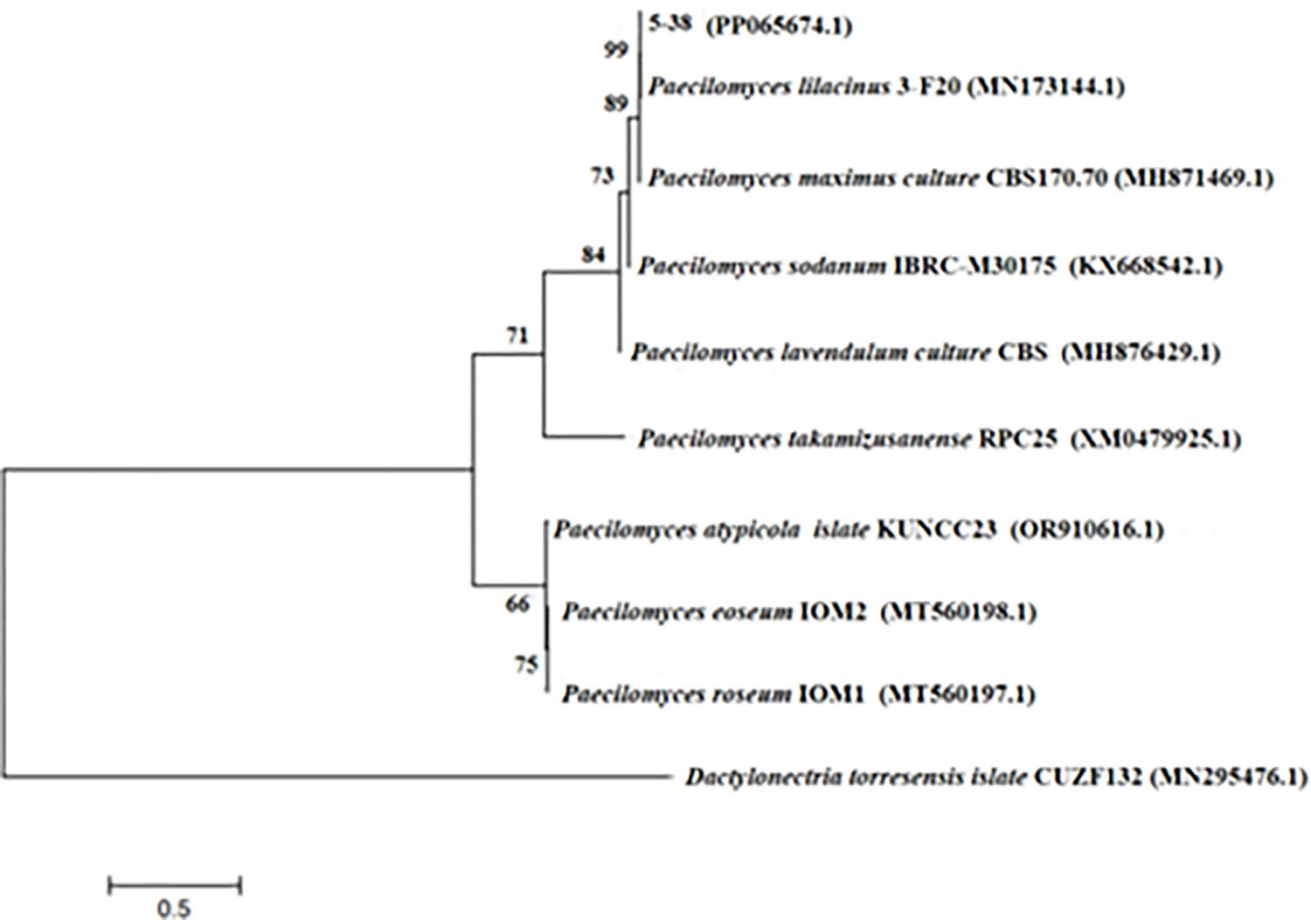
Phylogenetic tree of strain 5–38 based on ITS gene sequence.

Strains 5–38 were sequenced using ITS and the sequences were submitted to GenBank with the accession number PP065674. Analysis of the amplified sequences in the NCBI database revealed that that strains 5–38 exhibit up to 99% similarity to *Paecilomyces lilacinus*. A phylogenetic tree of the ITS genes was constructed using the MEGA11 software. (Fig 3) illustrates that strains 5–38 form a cluster within the same branch as *P*. *lilacinus*, indicating a recent affinity to *P*.*lilacinus*. Through a combination of morphological observations and molecular biology analysis, strains 5–38 were identified as *P*. *lilacinus*.

#### Determination of antibacterial spectrum

**Fig 4:**
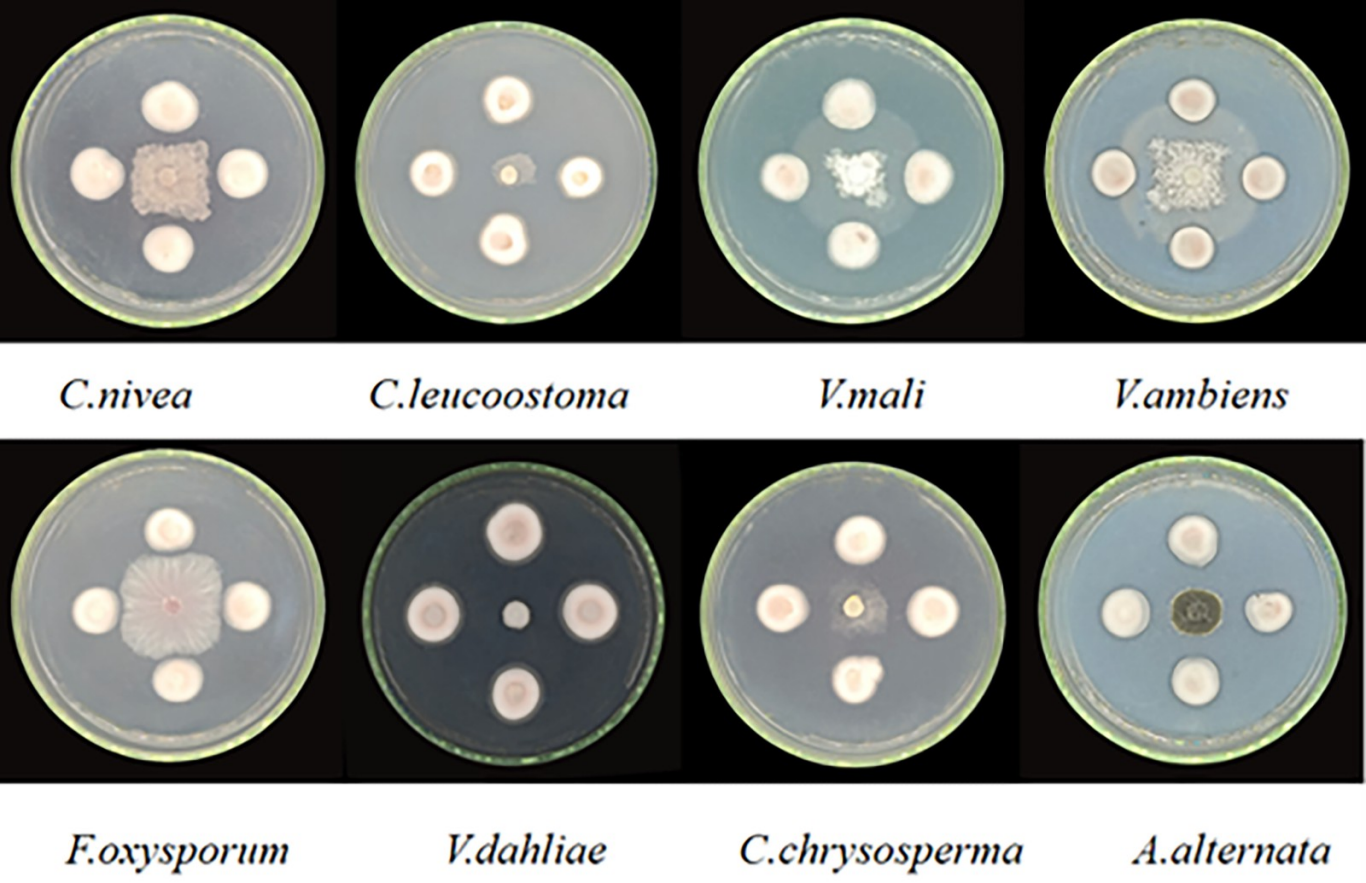
Mycelial inhibition of strains 5–38 against eight pathogens.

The inhibitory effect of antagonistic strain 5–38 on 8 tested pathogenic fungi was determined by cross plate confrontation method. As illustrated in Table 3, strain 5–38 effectively inhibited the growth of all 8 pathogens towards the periphery (Fig 4). The calculation of inhibition rates revealed that strain 5–38 inhibited 70% of the 8 pathogens (Table 4), exhibiting the highest efficacy against *C*. *leucostoma*, *V*. *mali*, and *V*. *dahliae* at 85.00%, 81.00%, and 81.00%, respectively. Additionally, *V*. *ambiens*, *C*. *chrysosperma*, and *A*. *alternata* were inhibited by more than 75%, specifically at 77%, 76%, and 79%, respectively. *C*. *nivea* and *F*. *oxysporum* showed inhibition rates of 73% and 72%, respectively.

**Table 3:** Inhibitory effects of strain 5–38 on eight pathogens.

Strains	Inhibition rate (%)
C.nivea	73.00±2.13e
C.leucostoma	84.50±2.10a
V. mali	81.21±1.15b
V. ambiens	77.41±1.47bc
F. oxysporum	72.04±0.71d
V. dahliae	81.40±1.13b
C. chrysosperma	76.52±1.07e
A. alternata	79.00±0.90bc

Note: The data listed in the table are mean ± standard deviation, and different lowercase letters represent whether the difference is significant in the <0.05.

**Table 4:** Inhibition of *C*. *chrysosperma* hyphae by different concentrations of antagonistic fungus.

Fermentation filtrate concentration	Inhibition ratio (%)
3%	65.83±1.31e
6%	71.96±0.79d
9%	86.92±1.53c
12%	89.31±0.60b
15%	92.28±0.41a

Note: The data listed in the table are mean ± standard deviation, and different lowercase letters represent whether the difference is significant in the <0.05.

### Effects of fermentation filtrate of different concentrations of biocontrol fungi on mycelia growth of *C*. *chrysosperma*

**Fig 5:**
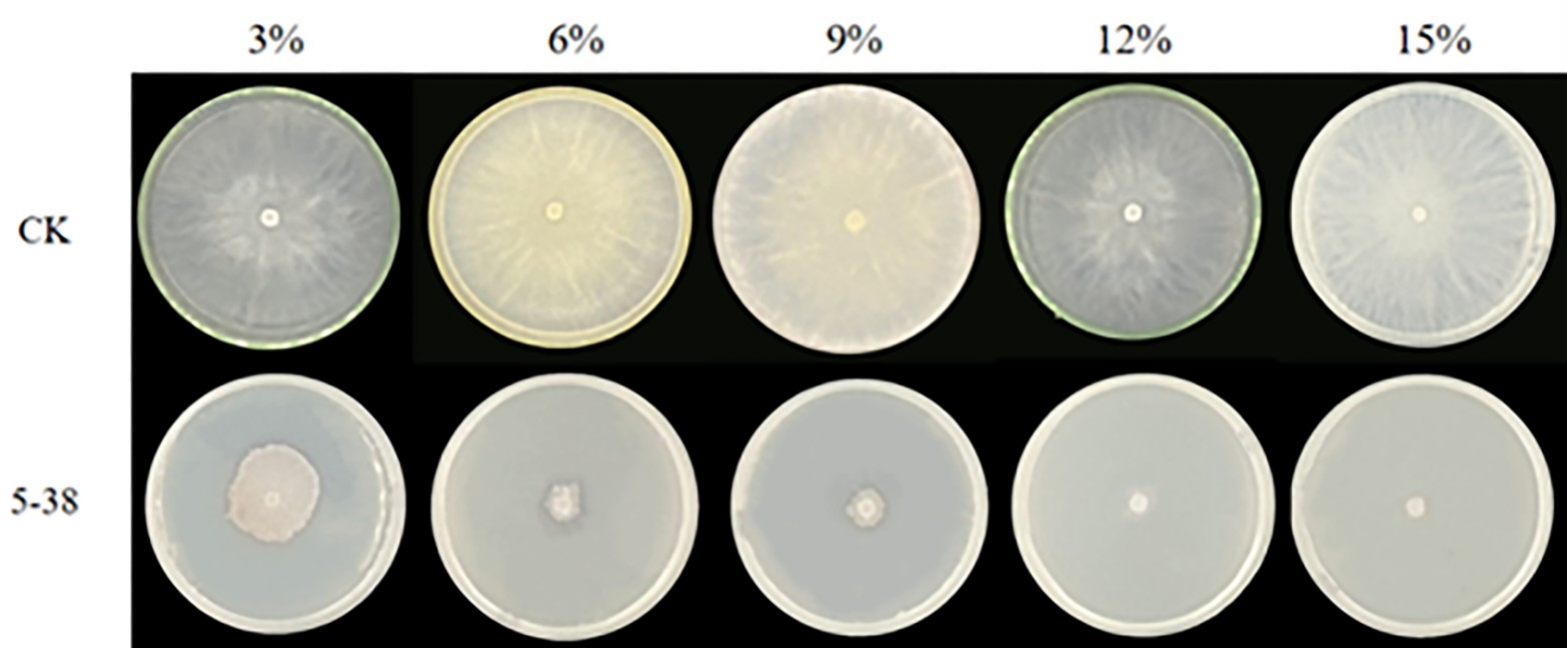
Effect of different concentration of fermentation liquid on mycelia growth of *C*. *chrysosperma*. CK = culture medium without the fermentation filtrate served as the control.

To investigate the inhibitory effect of *C*. *chrysosperma* mycelial growth, different proportions (3%, 6%, 9%, 12%, 15%) of fermentation filtrate were mixed with PDA medium. *C*. *chrysosperma* cakes were inoculated with strain 5–38 and incubated at a constant temperature of 26°C for 3 days. The inhibition of *C*. *chrysosperma* mycelium growth was evaluated using the mycelium growth rate method (Fig 5). Results indicated that strain 5–38 exhibited a significant inhibitory effect on *C*. *chrysosperma*, with the inhibition effect increasing as the fermentation filtrate concentration rose. Specifically, at a 3% concentration, strain 5–38 inhibited *C*. *chrysosperma* growth by 65.83%, while at a 15% concentration, it displayed the highest inhibition rate of 92.28% (Table 4).

#### Determination of thermal stability of biocontrol fungi

**Fig 6:**
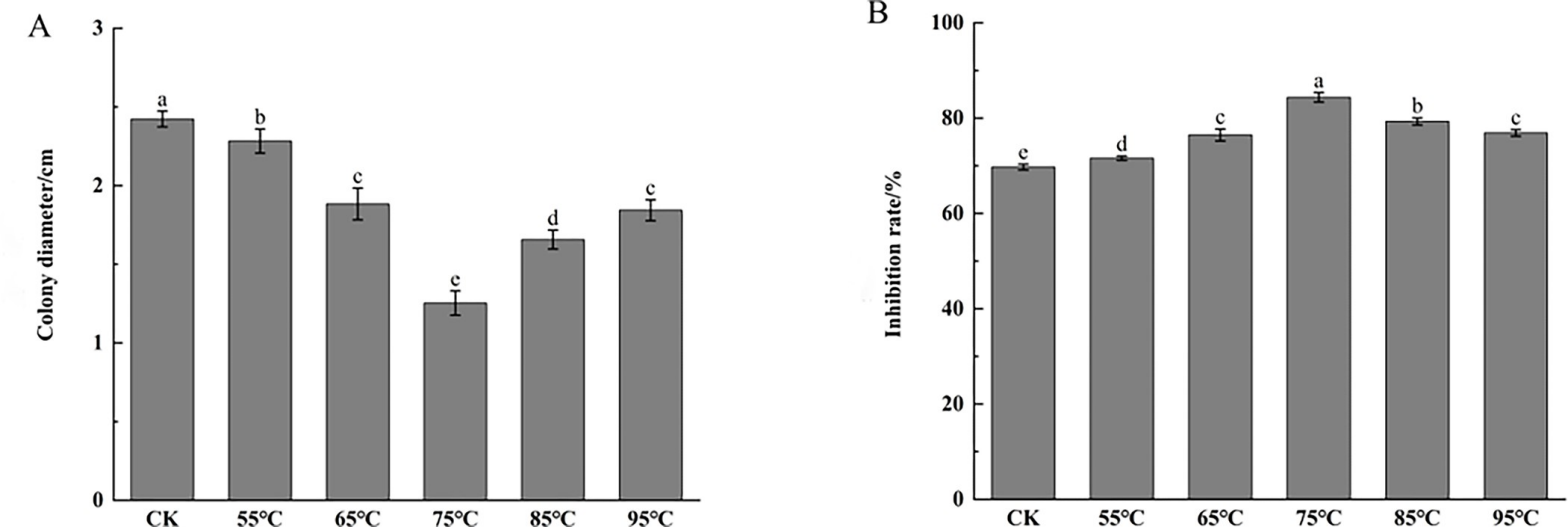
Effect of temperature on Fungal-inhibited activity of biocontrol fungi fermentation filtrate. A: Colony diameter of strain 5–38 after treatment; B: Inhibition rate of strain 5–38 after treatment. Note: Data are the average of three replicates and presented as the means±standard error. Different lowercase in the same column indicate significant differences at 0.05, respectively;CK = culture medium without the fermentation filtrate served as the control.

The results presented in Fig 6 demonstrate that the fermentation filtrate of strain 5–38, when exposed to a temperature of 75°C, exhibited the highest inhibition rate of *C*. *chrysosperma* growth at 84.63%. Furthermore, it was observed that as the treatment temperature exceeded 75°C, the inhibition rate started to decrease; however, it remained higher than that of the positive control (Fig 6A and 6B). These findings suggest that the fermentation filtrate of the 5–38 antagonistic fungi displayed increased antibacterial activity following temperature gradient treatment, highlighting the importance of maintaining an appropriate temperature to enhance the metabolites’ ability to inhibit pathogenic fungi.

#### Control of diseased spots in vitro branches of fermentation broth

According to Table 5, the biocontrol fungal fermentation was compared with the control group. The lesion area was 0.62 cm^2^, and biocontrol showed 88.44% effectiveness against *C*. *chrysosperma*.*C*. *chrysosperma*.

**Table 5:** Damage test of isolated branches by fermentation broth of antagonistic strains.

Strains	Lesion area (cm^2^)	Prevention effect (%)
CK	6.32±0.32a	-
5–38	0.73±0.05b	88.44%

Note: The data listed in the table are mean ± standard deviation, and different lowercase letters represent whether the difference is significant in the <0.05.

#### Effect of different concentration of fermentation filtrate on the growth of walnut seeds

**Fig 7:**
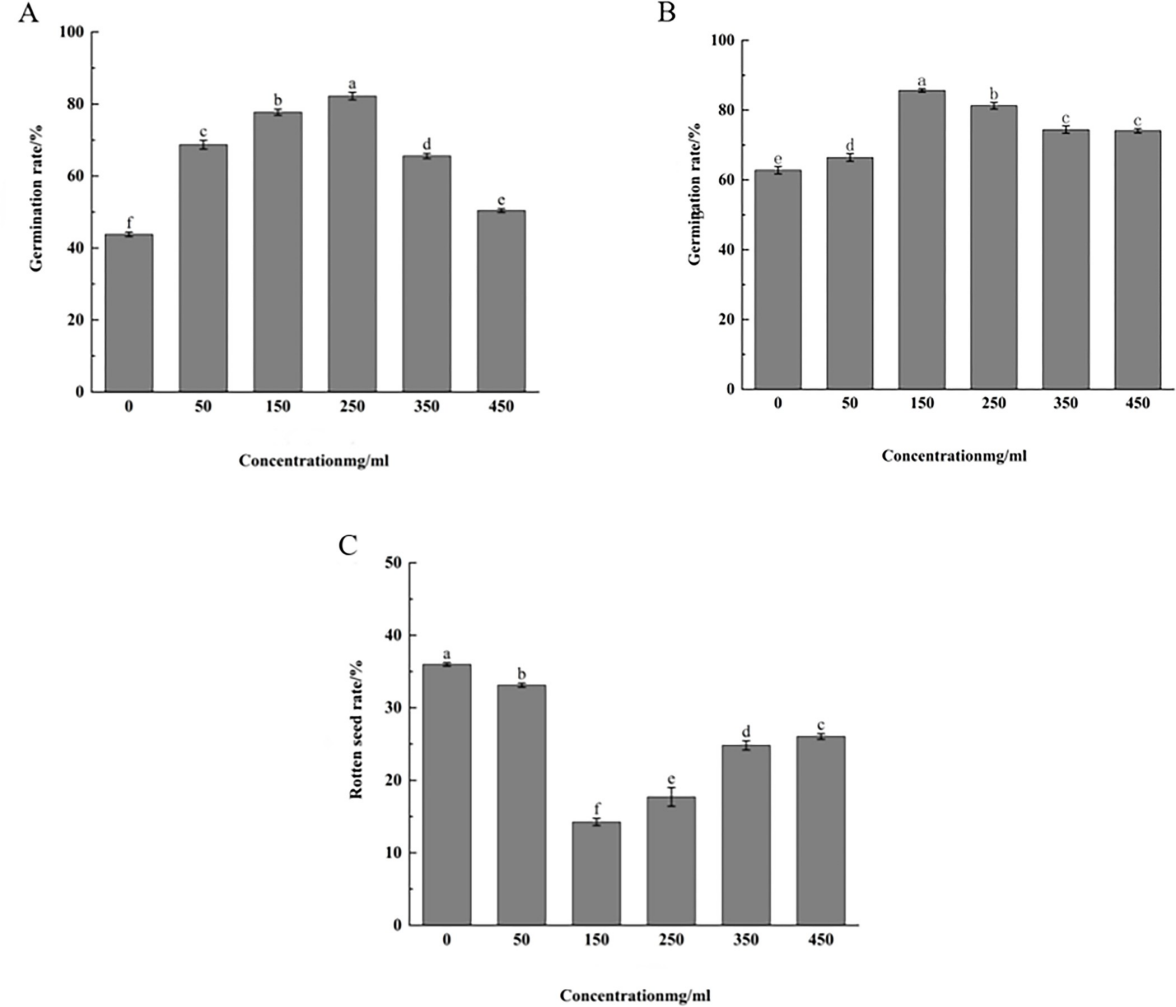
Effects of different fermentation filtrate concentration on germination rate, germination rate and bad seed rate of walnut seeds. A: Germination rate of walnut with shell after treatment of strain 5–38; B: Germination rate of walnut with shell after treatment of strain 5–38; C: Bad seed rate of walnut with shell after treatment of strain 5–38. Note: Data are the average ofthree replicates and presented as the means±standard error. Different lowercase in the same column indicate significant differences at 0.05, respectively;CK = culture medium without the fermentation filtrate served as the control.

The results presented in (Fig 7) indicate that the germination rate of walnut seeds significantly increases when treated with antagonistic fungal fermentation filtrate, particularly at concentrations of around 150 mg/ml and 250 mg/ml. The seed germination effect was observed to be highest at 250 mg/ml followed by 150 mg/ml, 50 mg/ml, 350 mg/ml, and 450 mg/ml, which was significantly higher than the control (P <0.05), The highest germination rates were recorded at 83.70% and 76.68% for concentrations of 250 mg/ml and 150 mg/ml, respectively (Fig 7A).

As illustrated in (Fig 7B), the germination rate of walnut seeds with the shell was influenced by the concentration of antagonistic fungi. The filtrate of *P*. *lilacinus* at concentrations between 50–150 mg/ml resulted in a gradual increase in the germination rate of walnut seeds, peaking at 150 mg/ml of fermentation. which gradually weakened when the concentration of fermentation filtrate exceeded 250 mg/ml. Therefore, It is evident that the concentration of the filtrate between 150 and 250 mg/ml significantly promoted walnut seed germination.

The study demonstrated that as the concentration of fermentation filtrate increased, the rate of poor seed planting for walnut seeds initially decreased before slightly increasing. (Fig 7C) Specifically, *P*.*lilacinus* 5–38 exhibited the lowest poor rate at 150 mg/ml, with a rate of 14.68%. Beyond a concentration of 150 mg/ml, the poor planting rate showed an upward trend, albeit still significantly lower than that of the control group (P < 0.05) after each concentration.

#### Different concentrations of fermentation filtrate on the growth of walnut seedlings

**Fig 8:**
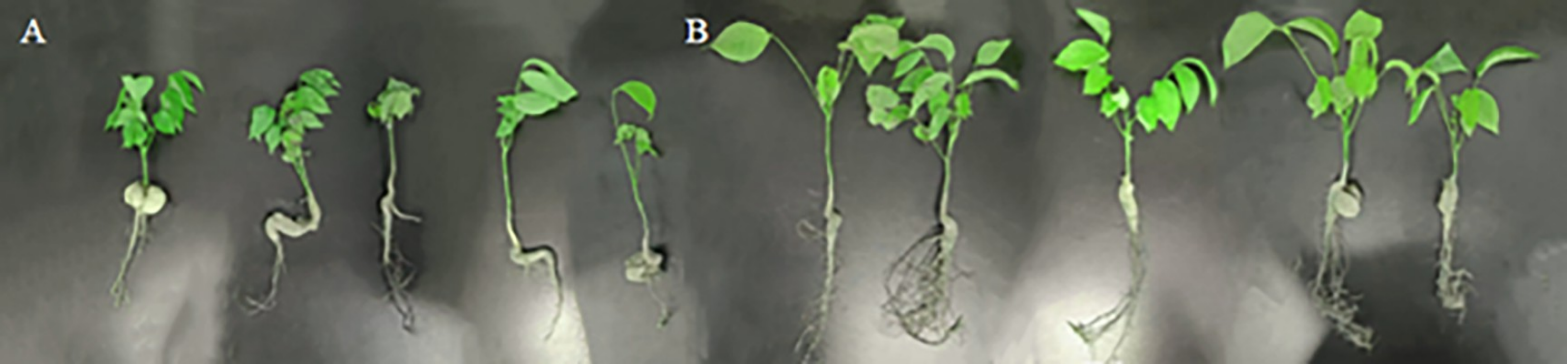
Growth promotion test of strain 5–38 on walnut seedlings. A: Contrast; B: Treated walnut seedlings with strain 5–38.

The antagonistic fungi fermentation filtrate exhibited optimal germination rates at concentrations of 150 mg/ml and 250 mg/ml. However, the incidence of non-viable seeds was markedly elevated at 250 mg/ml compared to 150 mg/ml. Consequently, the 150 mg/ml concentration was selected for subsequent promotion assays on walnut seedlings. Analysis of the data (Table 6) revealed that walnut seedlings treated with *P*. *lilacinus* 5–38 demonstrated a mean growth index of 12.63 cm, a primary root length of 26.67 cm, 16.37 lateral roots, a foliar area of 39.89 cm^2^, and a dry mass of 4.56 g (Fig 8). In contrast, control specimens exhibited a height of 9.70 cm, primary root length of 19.92 cm, 9 lateral roots, foliar area of 38.34 cm^2^, and dry mass of 2.30 g. The *P*. *lilacinus* 5-38-treated seedlings displayed significant enhancements in all measured parameters compared to the control group, with increases of 30.12%, 33.89%, 81.89%, 6.83%, and 98.26% in height, primary root length, lateral root quantity, foliar area, and dry mass, respectively.

**Table 6:** Effects of fermentation filtrate of strain 5–38 on growth indexes of walnut seedlings.

Strains	Plant height (cm)	Root length (cm)	Fibrous root (twig)	Blade area (cm^2^)	Dry weight (g)
ck	9.70±0.05b	19.92±0.09b	9.00±0.65b	37.34±0.43b	2.30±0.99b
5–38	12.63±2.13a	26.67±2.72a	16.37±3.30a	39.89±0.54a	4.56±0.77a
Increase rate%	30.21%	33.89%	81.89%	6.83%	98.26%

Note: The data listed in the table are mean ± standard deviation, and different lowercase letters represent whether the difference is significant in the <0.05.

## Discussion

The study elucidated a pronounced antagonistic effect of the fungus on walnut rot pathogens and various pathogenic fungi, culminating in localized expansion, rupture, and inhibition of hyphal and spore development. Dai et al. [17] demonstrated that antagonistic fungi substantially ameliorated rot disease. The isolated and screened Fungi 153 not only impeded apple rot hyphal growth but also suppressed spore germination. Moreover, the fungal fermentation broth exhibited inhibitory effects on the propagation of branch disease lesions. Shi et al. [18] observed that the *P*. *lilacinus* NH-PL-03 strain induced local expansion of *F*. *oxysporum* hyphae, increased cytoplasmic density, and cell wall degradation. While these findings align with the current study, the confrontation culture results suggested that *P*. *lilacinus* did not exhibit a significant growth advantage or clear reparasitism effect on pathogen hyphae.

Upon examining the inhibitory effect on *C*. *chrysosperma*, We ascertained that the efficacy of strain 5–38 filtrate intensified with increasing concentration. Thermal stability tests revealed robust inhibition at temperatures below 75°C, with diminished activity observed above this threshold. This observation suggests that metabolites produced by *P*. *lilacinus* 5–38 may experience reduced antibacterial activity or inactivation at elevated temperatures, although the strains still exhibited laudable thermal stability. These findings suggest that the fermentation filtrate of the 5–38 antagonistic fungi displayed increased antibacterial activity following temperature gradient treatment, highlighting the importance of maintaining an appropriate temperature to enhance the metabolites’ ability to inhibit pathogenic fungi.Wang et al. [9] elucidated that the antimicrobial effect of *P*. *lilacinus* strain 36–1 primarily stemmed from anti-biomass compounds produced during fermentation, with prolonged high-temperature treatments attenuating their potency. Li et al. [19] corroborated that inhibitory substances obtained from *P*. *lilacinus* fermentation exhibited poor tolerance to high temperatures. This study further corroborated that anti-biomass compounds are largely secreted into the fermentation filtrate during late-stage fermentation, and fungal-inhibitory substances can be isolated via centrifugation, albeit with limited high-temperature tolerance.

The fermentation filtrate also demonstrated a promotive effect on walnut seeds and seedlings, with optimal germination rates and lowest germination time observed at a concentration of 150 mg/mL. Noteworthy enhancements were noted in seedling height, primary root length, lateral root number, leaf area, and dry weight. Abdeldaym et al. [20] found that P. lilacinus secondary metabolites could regulate plant growth through the production of growth hormones. Liu et al. [21] reported that the fermentation broth, resembling IAA, promoted the growth of wheat coleoptiles and cucumber cotyledons. Xia et al. [22] observed growth promotion at low concentrations and inhibition of cabbage seed germination at high concentrations. These experimental outcomes align with our findings, suggesting the potential for further application of this antagonistic fungus.
